# Application of unsupervised deep learning algorithms for identification of specific clusters of chronic cough patients from EMR data

**DOI:** 10.1186/s12859-022-04680-4

**Published:** 2022-04-19

**Authors:** Wei Shao, Xiao Luo, Zuoyi Zhang, Zhi Han, Vasu Chandrasekaran, Vladimir Turzhitsky, Vishal Bali, Anna R. Roberts, Megan Metzger, Jarod Baker, Carmen La Rosa, Jessica Weaver, Paul Dexter, Kun Huang

**Affiliations:** 1 Indiana University School of Medicine, 1101 W 10th Street, Indianapolis, IN 46202 USA; 2 Purdue School of Engineering and Technology, IUPUI, ET 301L, 799 W. Michigan Street, Indianapolis, IN 46202 USA; 3 Center for Observational and Real-World Evidence, Merck & Co., Inc., Kenilworth, NJ USA; 4 Regenstrief Institute, Inc., Indianapolis, IN USA; 5 Eskenazi Health, Indianapolis, IN USA

**Keywords:** Chronic cough, Unsupervised learning, Deep clustering, EMR data

## Abstract

**Background:**

Chronic cough affects approximately 10% of adults. The lack of ICD codes for chronic cough makes it challenging to apply supervised learning methods to predict the characteristics of chronic cough patients, thereby requiring the identification of chronic cough patients by other mechanisms. We developed a deep clustering algorithm with auto-encoder embedding (DCAE) to identify clusters of chronic cough patients based on data from a large cohort of 264,146 patients from the Electronic Medical Records (EMR) system. We constructed features using the diagnosis within the EMR, then built a clustering-oriented loss function directly on embedded features of the deep autoencoder to jointly perform feature refinement and cluster assignment. Lastly, we performed statistical analysis on the identified clusters to characterize the chronic cough patients compared to the non-chronic cough patients.

**Results:**

The experimental results show that the DCAE model generated three chronic cough clusters and one non-chronic cough patient cluster. We found various diagnoses, medications, and lab tests highly associated with chronic cough patients by comparing the chronic cough cluster with the non-chronic cough cluster. Comparison of chronic cough clusters demonstrated that certain combinations of medications and diagnoses characterize some chronic cough clusters.

**Conclusions:**

To the best of our knowledge, this study is the first to test the potential of unsupervised deep learning methods for chronic cough investigation, which also shows a great advantage over existing algorithms for patient data clustering.

## Introduction

Chronic cough (CC), or cough lasting more than 8 weeks, affects approximately 10% of adults and is a common outpatient complaint. Affected individuals can cough hundreds or even thousands of times per day [1], seriously impairing their quality of life [2]. Common causes of CC include postnasal drip (upper airway cough syndrome), asthma, gastroesophageal reflux disease (GERD), infection of the respiratory system, chronic obstructive pulmonary disease (COPD), and angiotensin-converting enzyme (ACE) inhibitors [3]. Chronic cough can cause a variety of health problems such as sleep disruption, headache, and dizziness [4]. Chronic cough is often treated empirically, targeting common cause(s). Given the multi-factorial etiology of CC, many individuals with CC do not respond to treatment [5], highlighting the need to identify such individuals for both prospective and retrospective studies. Unlike other diseases, there is no ICD diagnosis code for CC, greatly increasing the difficulty of identifying and analyzing patients with this chronic disease.

Machine learning methods have been extensively used to analyze EMR data to predict and classify disease states [6, 7], model disease progression [8, 9], recommend interventions [8, 10], and predict future risks [11]. Most of this prior research has employed supervised learning algorithms, commonly using diagnosis codes as labels. But certain diseases, such as CC, do not have a corresponding ICD code. While annotating and labeling can be performed through chart review, such review is costly and time-consuming given the large numbers necessary to train machine learning algorithms.

One potential solution is to employ unsupervised clustering analysis to uncover subgroups within clinical data [12]. Unsupervised learning requires no labels and targets to find structure within the data so that it can discover the hidden patterns in data or learn features towards the pattern discovery. This study continues previously conducted by Weiner et al. [13], which identified cough and CC patients using a rule-based algorithm. We apply deep learning-based unsupervised learning algorithm—deep clustering with the auto-encoder embedding (DCAE) on the data to identify clusters within the cough patient population and investigate characteristics of clusters dominated by CC patients. The results show that the deep learning-based algorithms have superior performance than the traditional k-means algorithm. The statistical analysis shows that unsupervised learning discovers some diagnoses and medications linking to chronic cough that match what has been discussed in the literature. To the best of our knowledge, this study is the first to test the potential of unsupervised deep learning methods for chronic cough investigation, which also shows a great advantage over the existing algorithms for patient data clustering.

## Study cohort

Using EMRs of a large statewide academic health system and a public county hospital, we constructed two cohorts: (1) patients with CC and (2) patients with a cough who did not meet the CC criteria. We extracted patients with cough aged 18–85 years with at least one outpatient visit between 10-01-2005 and 09-30-2015.

The CC [13] cases are defined as a patient who met the following two criteria: (1) at least three instances of cough on at least three separate visits within a 120-day window, which is the study period; (2) first and last instances of cough were required to span at least 8 weeks within the study period. The non-chronic cough patients presumably had an acute or subacute cough but did not meet the chronic cough criteria. In total, we identified 25,881 chronic cough (CC) patients and 238,265 non-chronic cough (Non-CC) patients from the EMR. Table 1 provides the demographic summary of the cohorts. Among these patients, 15,285 chronic cough patients had matched diagnoses, medications, and lab test data. So, we randomly selected the same number from the non-chronic cough patients with these structured data for the analysis. There is a total of 30,570 patients in the analysis cohort.

**Table 1:** Summary of the overall cohort

	Category	Non-CC (N = 238,265)	CC (N = 25,881)
Age	Mean (SD)	45.29 (17.81)	54.7 (16.35)
Gender	Male	91,768 (38.52%)	8985 (34.72%)
	Female	146,491 (61.48%)	16,896 (65.28%)
	Unknown	6	
Race	Black	48,864 (20.51%)	4674 (18.06%)
	Other	43,606 (18.3%)	1144 (4.42%)
	White	145,795 (61.19%)	20,063 (77.52%)
Urbanicity	Rural	21,181 (9.34%)	2848 (12.13%)
	Urban	205,484 (90.66%)	20,635 (87.87%)
	Unknown	11,600	2398

## Results

### Generating clusters using both non-CC and CC data

We first applied the deep clustering algorithm with auto-encoder embedding (DCAE) algorithm to generate clusters by using both Non-CC and CC data. The compared methods include: (1) K-means Clustering: In order to fairly compare with other methods, we firstly apply the PCA algorithm to reduce the dimensionality of the original data to 256, then use the K-means algorithm for clustering. (2) Hierarchical agglomerative clustering (HC): The hierarchical agglomerative clustering algorithm introduced in [14]. (3) Consensus clustering (CC): The consensus clustering algorithm introduced in [15]. (4) Deep AutoEncoders (DAE): Utilized the Deep AutoEncoder for feature extraction followed by the K-means algorithm for clustering. (5) Deep Clustering (DC): A variant of DCAE which overlooks the reconstruction loss. We used two metrics, namely Purity score [16] and Silhouette Value [17], to compare the performance of different clustering methods. Here, the Silhouette coefficient is a widely used metric for evaluating clustering results ranging from − 1 to 1 with 1 being the best. Purity Score indicates the extent of the generated clusters that are dominated by one category (CC or Non-CC) with 1 being the best.

**Fig. 1:**
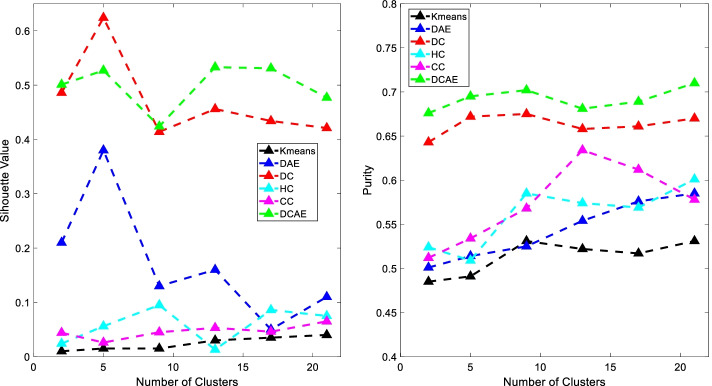
The purity and silhouette values of different methods, where the number of clusters k are varied from {2, 5, 9, 13, 17, 21}

**Fig. 2:**
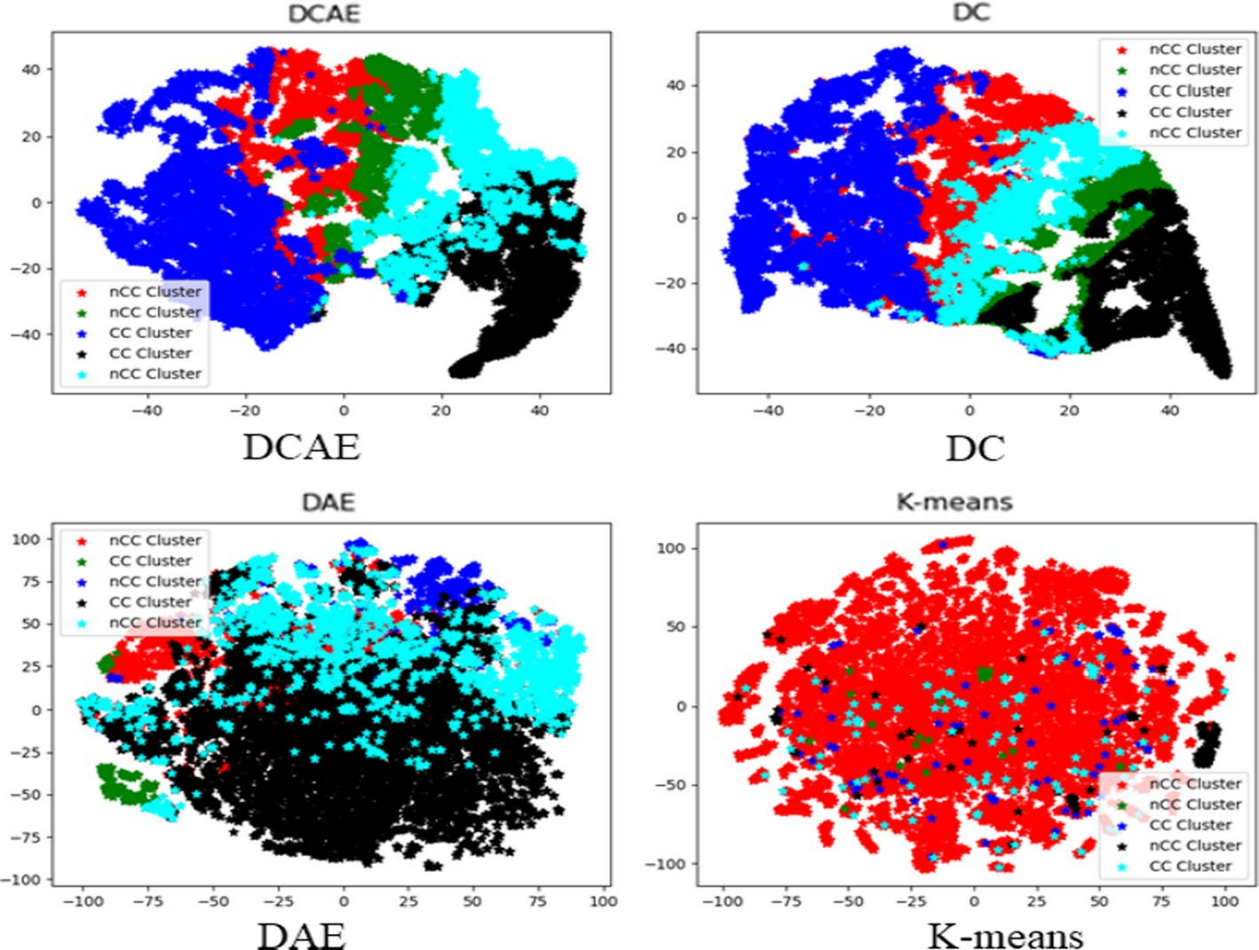
Visualization of the clustering results by tSNE [18]. Different colors represent different clusters. CC and Non-CC clusters correspond to CC and Non-CC patient dominant clusters

Figure 1 shows that, (1) The deep clustering-based methods (i.e., DC, DCAE) consistently work better than the K-means, HC, CC and DAE methods in terms of Silhouette value and Purity score. The reason being that the DC method is an end-to-end clustering framework that can learn specific features that are useful for the clustering task, and the tSNE plots [18] shown in Fig. 2 could also demonstrate the advantage of the DC and DCAE methods for separating cough patients into different clusters. (2) DCAE can achieve superior Purity scores than DC due to the consideration of the local structure of the data. These results indicate that another advantage of our DCAE algorithm is that it could more effectively separate CC patients from Non-CC patients than the DC algorithm.

Finally, we also found that the number of CC dominated clusters (which means over 50% of the instances in a cluster are CC) are more than Non-CC dominated clusters (shown in Table 2). One possible reason is that the CC patients may have more subtypes in comparison with the Non-CC patients, and our DCAE has the potential to stratify CC patients into different phenotypes that can benefit their personalized treatment design.

**Table 2:** The number of CC dominated clusters (nCCD) for our DCAE method

Cluster number	K = 5	K = 9	K = 13	K = 17	K = 21
nCCD	3	7	11	14	18

### Influence of the parameters in DCAE model

**Fig. 3:**
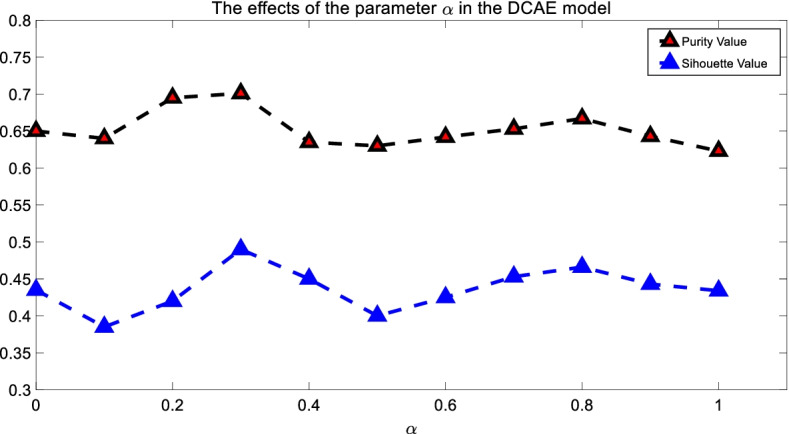
The influence of the parameter $\alpha$ in the DCAE model

Our DCAE model (shown in Eq. (2)) uses the parameter α to balance the importance of the clustering and reconstruction loss. In this section, the influence of α in the DCAE workflow is discussed. Here, we fixed the cluster number as 21 and the results are shown in Fig. 3, where α varies in the range [0,1] with interval 0.1. As can be seen from Fig. 3, for both the purity and Silhouette values, most inner intervals of the curve have larger values (i.e., better clustering performance) than the leftmost and rightmost vertices, which indicate the effectiveness of combining both clustering and reconstruction loss for identifying cough patients’ phenotypes. Moreover, the higher performance (when α equals 0.3) implies each loss function in the DCAE model has its contribution for achieving good clustering performance.

### Analysis of the non-CC versus CC clusters

By applying the DCAE algorithm, we identified four clusters with high purity values from the results when the number of clusters is set to 21. Three of them are CC clusters (shown in Table 3). Among these three CC clusters, CC-1 is the largest with 4658 CC instances. The other two CC clusters, named CC-2, CC-3, respectively, are relatively small, with 1737 and 154 instances. The percentages of CC instances in three CC clusters are 83.8%, 73.8%, and 70.0%, respectively. The non-CC cluster has 73.1% non-CC instances. We compared the CC instances in the CC clusters against the Non-CC instances within the Non-CC cluster using categorized diagnosis, medication, and lab tests of interest, such as blood count, sputum culture, Arterial Blood Gas Test (ABG), by categorizing them based on Phecode, internal medication, and lab test categorization, and the results are listed in Table 3. The values in Table 3 show the percentage of patients who have the corresponding diagnosis, medication, or lab tests. Comparing the CC clusters against the Non-CC cluster, we found that about 90% of the patients in the CC clusters have respiratory diagnoses, whereas 23.32% of patients in the Non-CC cluster have respiratory diagnoses, reflecting the nature of the CC disease since it is linked to different respiratory issues. We also found that more than 55% of patients in the CC clusters have either circulatory system diseases or endocrine disorders, whereas less than 20% of patients in the Non-CC cluster have either of these two diagnoses.

**Table 3:** Univariate analysis of the categorized diagnosis, medication, and lab data of the non-CC and CC clusters

	Non-CC (N = 8588)	CC-1 (N = 4658)	CC-2 (N = 1737)	CC-3 (N = 154)	*p* value
*Diagnosis*					
Respiratory	2003 (23.32%)	4300 (92.31%)	1593 (91.71%)	140 (90.91%)	< .0001
Endocrine metabolic	1268 (14.76%)	3514 (75.44%)	1026 (59.07%)	88 (57.14%)	< .0001
Circulatory system	1687 (19.64%)	3735 (80.18%)	1110 (63.9%)	91 (59.09%)	< .0001
Mental disorder	1020 (11.88%)	3133 (67.26%)	857 (49.34%)	56 (36.36%)	< .0001
Neurological	805 (9.37%)	2381 (51.12%)	579 (33.33%)	38 (24.68%)	< .0001
Digestive	1419 (16.52%)	2889 (62.02%)	767 (44.16%)	60 (38.96%)	< .0001
Symptoms	2344 (27.29%)	3335 (71.6%)	943 (54.29%)	71 (46.1%)	< .0001
Hematopoietic	336 (3.91%)	2353 (50.52%)	484 (27.86%)	20 (12.99%)	< .0001
*Medication*					
Antiasthmatic bronchodilator	1099 (12.8%)	2777 (59.62%)	898 (51.7%)	79 (51.3%)	< .0001
Minerals electrolytes	1164 (13.55%)	2791 (59.92%)	704 (40.53%)	52 (33.77%)	< .0001
Corticosteroids	1035 (12.05%)	2529 (54.29%)	728 (41.91%)	61 (39.61%)	< .0001
Ulcer drugs	1714 (19.96%)	2789 (59.88%)	769 (44.27%)	55 (35.71%)	< .0001
*Lab*					
Blood count	3437 (40.02%)	4078 (87.55%)	1287 (74.09%)	105 (68.18%)	< .0001

The *p* values for the comparison are bracketed in the last column. Only < .0001 was listed in the last column if all *p* values were < .0001

In addition, all three CC clusters have a higher percentage of symptom diagnosis than the Non-CC cluster, which is 27.29%. The diagnosis category symptoms include symptoms, signs, abnormal results of clinical or other investigative procedures, and ill-defined conditions regarding which no diagnosis classifiable elsewhere is recorded. Specifically, the symptoms include ‘R05 cough’ and ‘R06.0 Dyspnea’, related to CC. Except for CC-3 (46.1%), CC-1 and CC-2 have more than 50% of the patients having symptom diagnoses. In addition, over or close to 50% of the patients in CC-1 and CC-2 have mental disorders. The smallest cluster (CC-3) also has 36.36% of patients having mental disorders as a diagnosis. Only 11.88% of patients in the Non-CC have mental disorder diagnoses.

From the medication aspect, more than 50% of the patients in the CC clusters were prescribed antiasthmatic and bronchodilator medications, whereas only 12.8% of Non-CC patients in the analysis cohort were prescribed these medications in this category. About 40% of the patients in the CC clusters take corticosteroids, whereas only 12.05% of the Non-CC patients take this type of medication. These three types of drugs are often used to treat patients with CC [19–21], chronic nonproductive cough [22], or CC due to asthma [23]. There are 59.88%, 44.27%, 35.71% patients in cluster CC-1, CC-2, and CC-3 taking ulcer drugs, whereas only about 19.96% of the patients in the Non-CC cluster taking ulcer drugs. In addition, 68.18–87.55% of patients in the CC clusters have had blood count lab testing, whereas only about 40.02% of the patients in the Non-CC cluster had blood count lab testing.

Given that CC is mainly a type of respiratory disease and the percentage of respiratory diagnoses of each CC cluster is over 90%, and there is no specific ICD code for CC, we further analyzed the significance of each diagnosis under this respiratory category to investigate whether some of the diagnoses are more used for or related to CC. Table 4 shows the respiratory category diagnoses with a significant difference (*p* value < 0.0001) between the Non-CC and CC clusters. We only listed the diagnoses that have over 10% of CC patients in any CC cluster to demonstrate the significance of those diagnoses on CC patients. Over 50% of patients in the CC cluster have a diagnosis of ‘Cough’, whereas only 3.76% of the patients in the Non-CC cluster have a diagnosis of ‘Cough’. The diagnoses of ‘Shortness of Breath’ and ‘Other Dyspnea’ are more than 15% in any CC cluster, but only less than 2% in the Non-CC cluster.

**Table 4:** Univariate analysis on respiratory diagnosis between CC and non-CC clusters

	Non-CC (N = 8588)	CC-1 (N = 4658)	CC-2 (N = 1737)	CC-3 (N = 154)	*p* value
Chronic airway obstruction	82 (0.95%)	1376 (29.54%)	318 (18.31%)	25 (16.23%)	< .0001
Obstructive chronic bronchitis	41 (0.48%)	741 (15.91%)	178 (10.25%)	17 (11.04%)	< .0001
Cough	323 (3.76%)	2503 (53.74%)	1001 (57.63%)	79 (51.3%)	< .0001
Pneumonia	99 (1.15%)	1143 (24.54%)	279 (16.06%)	13 (8.44%)	< .0001
Shortness of breath	121 (1.41%)	1156 (24.82%)	323 (18.6%)	23 (14.94%)	< .0001
Other dyspnea	148 (1.72%)	1296 (27.82%)	295 (16.98%)	24 (15.58%)	< .0001
Asthma	230 (2.68%)	716 (15.37%)	284 (16.35%)	21 (13.64%)	< .0001
Other diseases of lung	55 (0.64%)	559 (12%)	173 (9.96%)	7 (4.55%)	< .0001
Acute bronchitis and bronchiolitis	99 (1.15%)	597 (12.82%)	248 (14.28%)	19 (12.34%)	< .0001
Respiratory Failure	18 (0.21%)	568 (12.19%)	86 (4.95%)	3 (1.95%)	< .0001
Pleurisy pleural effusion	27 (0.31%)	533 (11.44%)	102 (5.87%)	6 (3.9%)	< .0001

We further analyzed the significance of diagnoses under categories circulatory system diagnosis or endocrine disorders to investigate whether some of the diagnoses are more related to CC, with the results are shown in Tables 5 and 6. From Tables 5 and 6, we observe the diagnoses with a significant difference between the Non-CC cluster and CC clusters, and one of the CC clusters has over 10% of patients with the diagnosis. The largest CC cluster (CC-1) has over 35% of patients that have hyperlipidemia, and even the smallest CC cluster (CC-3) has 14.94% patients with hyperlipidemia. In contrast, the percentage of patients with hyperlipidemia in the Non-CC cluster is only 2.28%. Similarly, the Non-CC cluster has only 4.1% of patients with type 2 diabetes, whereas the CC clusters have 13.64–27.31% of patients with type 2 diabetes. The CC clusters also have higher percentages of obesity, hypothyroidism, and hypovolemia patients. Table 5 shows the p-values for all the differences are < 0.0001, except the difference between CC-3 and Non-CC on hypovolemia, which is not significant (0.9293). Table 6 also indicates that the largest CC cluster (CC-1) has over 51% of patients having essential hypertension. The other two CC clusters have 27.92% and 37.25% of patients with essential hypertension, whereas less than 10% of the patients in the Non-CC cluster have essential hypertension. The three CC clusters have over or close to 20% of patients diagnosed with nonspecific chest pain, whereas the Non-CC cluster has only 5.8%. The Non-CC cluster has very few (less than 0.5%) patients taking aspirin long term or currently or diagnosed with congestive heart failure or atrial fibrillation or hypertensive chronic kidney disease. However, the largest CC cluster has over 10% of patients with one or more of these diseases. Even the two small CC clusters have 1.3% to 5.07% of patients having these diseases. The p-values in Table 6 show that the differences between these percentages are significant.

**Table 5:** Univariate analysis on endocrine and metabolic diagnosis between CC and non-CC clusters

	Non-CC (N = 8588)	CC-1 (N = 4658)	CC-2 (N = 1737)	CC-3 (N = 154)	*p* value
Obesity	99 (1.15%)	491 (10.54%)	103 (5.93%)	7 (4.55%)	< .0001
Type 2 diabetes	352 (4.1%)	1272 (27.31%)	294 (16.93%)	21 (13.64%)	< .0001
Hyperlipidemia	196 (2.28%)	1650 (35.42%)	423 (24.35%)	23 (14.94%)	< .0001
Hypothyroidism	153 (1.78%)	713 (15.31%)	184 (10.59%)	12 (7.79%)	< .0001
Hypovolemia	51 (0.59%)	569 (12.22%)	78 (4.49%)	1 (0.65%)	(< .0001, < .0001, 0.9293)

The *p* values are shown in the last column. < .0001 was listed in the last column if all of three *p* values were < .0001

**Table 6:** Univariate analysis on circulatory system diagnosis between CC and non-CC clusters

	Non-CC (N = 8588)	CC-1 (N = 4658)	CC-2 (N = 1737)	CC-3 (N = 154)	*p* value
Essential hypertension	735 (8.56%)	2400 (51.52%)	647 (37.25%)	43 (27.92%)	< .0001
Nonspecific chest pain	498 (5.8%)	1201 (25.78%)	330 (19%)	35 (22.73%)	< .0001
Long term or current use of aspirin	8 (0.09%)	694 (14.9%)	84 (4.84%)	4 (2.6%)	< .0001
Congestive heart failure	27 (0.31%)	674 (14.47%)	88 (5.07%)	4 (2.6%)	< .0001
Atrial fibrillation	25 (0.29%)	572 (12.28%)	79 (4.55%)	2 (1.3%)	(< .0001, < .0001, 0.0255)
Hypertensive chronic kidney disease	14 (0.16%)	537 (11.53%)	59 (3.4%)	4 (2.6%)	< .0001

## Discussions

In this study, CC patients were extracted from the EMR using the rule-based algorithms developed by Weiner et al. [13]. Given the heterogeneity among the patients and disease etiology, to understand the structure of the patient data and subgroups of the CC patients, unsupervised learning can be applied. As demonstrated in this research, when we carried out unsupervised clustering of the patients using a deep learning-based approach, we were able to identify different clusters that are highly enriched with CC patients with different diagnoses. We found that (1) The CC patients have a higher percentage of chronic airway obstruction, pneumonia, and asthma diagnoses, which is consistent with the literature [24, 25]. As per CHEST guideline, asthma, gastroesophageal reflux disease (GERD), nonasthmatic eosinophilic bronchitis, and Upper Airway Cough Syndrome (UACS) are the most common causes of chronic cough. The guideline also suggests that initial empirical treatment based on clinical diagnosis of chronic cough should be considered as a more targeted approach [26]. Our result also shows that more chronic patients have a diagnosis of acute bronchitis and bronchiolitis. Although some literature [27] investigated CC related to acute viral bronchiolitis in children, our results shed light on future research to investigate the association between acute bronchiolitis and CC in adults. (2) More CC patients have obesity and type 2 diabetes. This result is consistent with one of the most recent findings in the literature [28], which found that CC seems to be more severe in obese patients and that diabetes may also be involved in the development of CC. Patients with obesity are more likely to develop hyperlipidemia, which might explain why CC patients have a higher rate of hyperlipidemia in the present study. (3) The CC patients have more hypothyroidism diagnoses, which is reflected in the study of Birring et al. [29, 30]. It might be associated with the idiopathic CC that is predominantly female. (4) Over 25% of CC patients in the CC clusters have essential hypertension. We thought this might be related to ACE inhibitors taken by the patients diagnosed with essential hypertension. Many research show ACE inhibitors are associated with CC [31]. Although during the 120-day timeframe, all patients in the study cohort did not take the ACE inhibitors. Some might take before the index date. (5) Over 19% of the CC patients within the three CC clusters have chest pain diagnoses, which might be associated with symptoms of some CC, such as gastroesophageal reflux‐related cough [32] or excessive coughing that might lead to chest pain.

Comparison across different CC clusters reveals significant differences in comorbidities and use of medications. These significant differences across different CC clusters might be due to the underlying heterogeneity in CC development in different patients. A better understanding of the causes of underlying heterogeneity in CC patients and disease etiology can be helpful in the development of effective treatment strategies and individualized patient care. In this research, due to a large number of features, the unsupervised learning methods are based on the clinical features of diagnosis only. Other structured data, such as medication and lab tests, were not explicitly utilized for the unsupervised learning even though we showed that there are significant differences in medications among these clusters. These observations suggest that there is potential to improve the clustering performance if medication and lab test results are utilized for cluster generation. On the other hand, the high dimensionality of the features will require more innovations in integrative clustering algorithms that are robust to noise. Furthermore, since CC is a syndrome that is not coded, the unstructured clinical notes may contain more information that can be used. This is the first study to apply deep learning based unsupervised learning approach to characterize CC using EMR data. This study suggests that deep learning-based unsupervised learning can be used in payer databases to estimate the burden of CC and the allocation of healthcare resources. Currently, we focus on structured data. However, we are carrying out a parallel study on extracting features from the clinical notes of this patient cohort which will allow further comparison of the contribution of unstructured data in patient clustering and stratification.

## Conclusions

In this paper, we developed and evaluated deep learning-based unsupervised learning algorithms to identify clusters of CC patients. The proposed DCAE deep learning method could better aggregate subjects into different groups than other methods while preserving the intrinsic structure of data and minimizing the clustering loss to manipulate the embedded feature space appropriately. We applied statistical analyses on the diagnoses data within the EMR to characterize and compare the CC and non-CC patient clusters identified using the DCAE. The results indicate that we can identify characteristics of patients within different CC clusters and between CC and non-CC clusters. This is the first study to apply deep learning based unsupervised learning approach to characterize CC using EMR data. Because CC is associated with other chronic conditions, for future work, further analysis will be carried out to extract the sub-clusters within the large CC cluster by considering the combination of diagnosis, medication, and lab tests, as well as potential information from unstructured clinical notes.

## Methods

### Feature engineering and data representation

For the current study, we utilized the structured data within the EMR to explore the clusters of CC and Non-CC cases, defined as positive instances of cough encounters within each patient's study period. Patient features were constructed from the medical history of the study period. In this study, we used the diagnoses as clinical features to generate clusters of patients for unsupervised learning algorithms. After the clusters are identified, diagnoses, medication, and lab tests are used to evaluate the differences among the patients. The patient diagnosis in our data is encoded with the International Classification of Diseases (ICD-10) codes. Considering a large number of distinct ICD codes, we used the first three digits of ICD-10. There is a total of 1228 unique three-digit ICD-10 codes in the study cohort. To construct the data representations, we applied Bag-of-Word [33], a natural language processing (NLP) text representation method, by treating each ICD code's occurrence as a word and a patient's records as a text document. Each patient was represented by the frequency of each ICD code in the medical history of the patient (i.e., term frequency also known as TF).

### Deep AutoEncoder

**Fig. 4:**
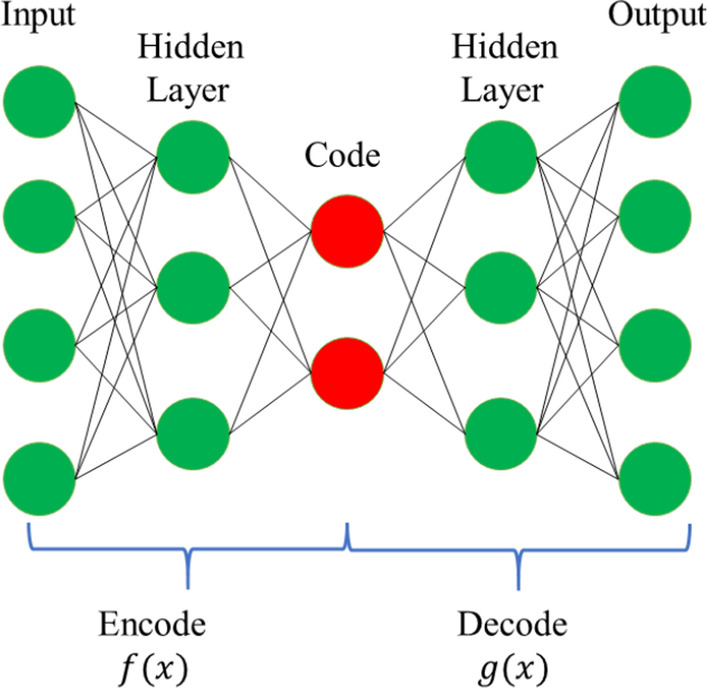
The framework for deep AutoEncoders (DAE)

Our clustering method is based on Deep AutoEncoders (DAE) framework [34]. Generally, DAE is used to learn a new representation of the input data to reduce their dimensionality. As shown in Fig. 4, the DAE is comprised of two parts, i.e., the encoder network *f* (*.*) and decoder network *g*(.) where the encoder network aims at compressing the data into the hidden space with lower dimensionality and the output network is exploited to reconstruct the original input from the hidden layer data. More specifically, given the input data *X* = (*x*_1_*, x*_2_*, …x*_*N*_)^*T*^ $\in$ *R*^*N*×*d*^, the objective function of DAE can be formulated as follows:

1 $$\frac{1}{N}\mathop \sum \limits_{i = 1}^{N} \left\| {g\left( {f\left( {x_{i} } \right)} \right) - x_{i} } \right\|_{2}^{2}$$

As shown in Eq. (1), DAE aims to find a code for each input sample by minimizing the mean squared errors between its input and output overall samples. Then, the latent representation $f\left( {x_{i} } \right)$ with lower dimensionality will take on the main information of the input data, and we can use them for the following learning task in order to capture the intrinsic structure of the data.

### Deep clustering with AutoEncoder embedding

**Fig. 5:**
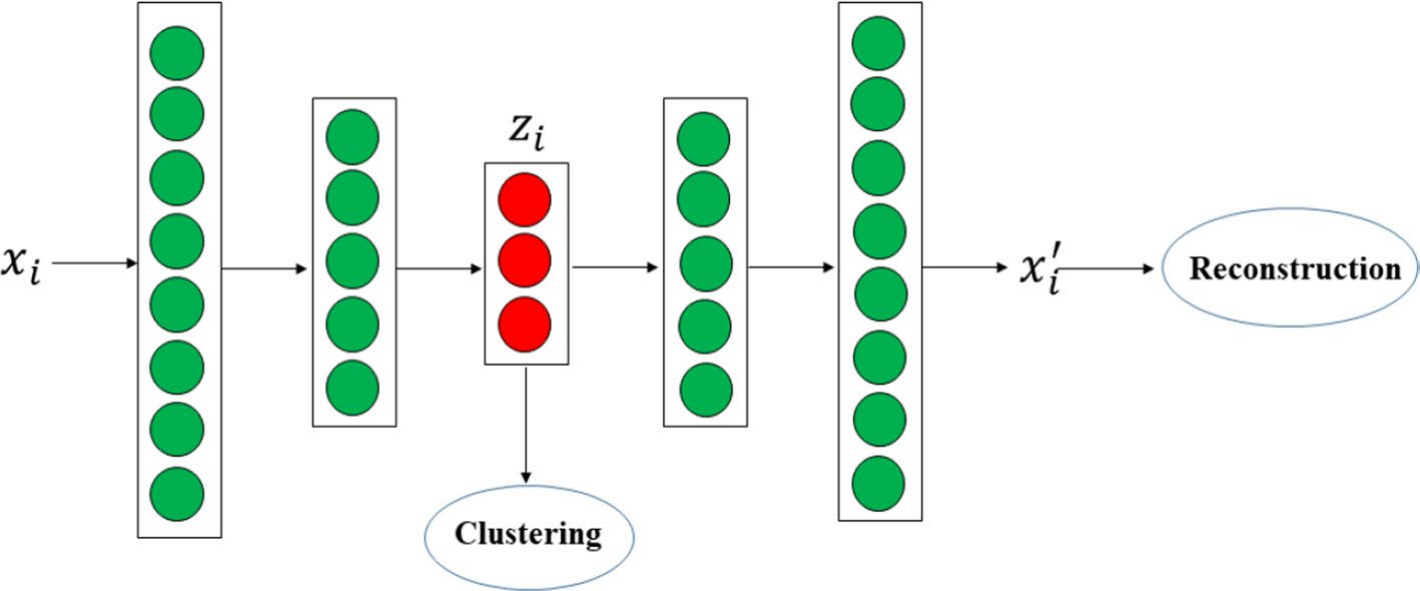
The framework for deep clustering with auto-encoder embedding (DCAE)

As discussed in the previous section, the DAE framework can learn compressed but meaningful encoding of the input data. Based on the DAE, we proposed a novel end-to-end deep clustering algorithm with auto-encoder embedding (DCAE) to aggregate the cough patients into different clusters. As shown in Fig. 5, the DCAE structure is composed of DAE and a clustering function connected to the embedding layer of DAE. More specifically, the encoding network of DAE consists of three fully connected layers and the node number in each layer is 1024, 512, and 256, respectively. We also adopted batch normalization (BN) after each hidden layer and before sigmoid activation in order to avoid the vanishing gradient problem. For each sample $x_{i} \in R^{d}$, we denote its corresponding code and reconstruction vector as $z_{i} \in R^{v}$ and $x_{i}^{^{\prime}} \in R^{d}$, respectively. Let $\mu_{j} \left( {j = 1, 2,3, \ldots , K} \right)$ be the center of cluster $C_{j}$ in the embedding space, and K represents the number of clusters to be obtained, then the objective function of DCAE can be formulated as follows:

2 $$L = \mathop \sum \limits_{i = 1}^{N} \mathop \sum \limits_{j = 1}^{K} I\left( {z_{i} ,u_{j} } \right)\left\| {z_{i} - u_{j} } \right\|_{2}^{2} + \alpha \mathop \sum \limits_{i = 1}^{N} \left\| {x_{i} - x_{i}^{^{\prime}} } \right\|$$

where $I\left( {z_{i} ,u_{j} } \right)$ is an indicator function, it equals 1 if $u_{j}$ is the center with the shortest distance to the data point $z_{i}$ among all the cluster centers, otherwise $I\left( {z_{i} ,u_{j} } \right) = 0$. From Eq. (2), we can observe that the first term is the clustering loss function, which aims to learn better embedding $z_{i}$ from the original data to minimize the distances among the subjects in the same cluster. The second term is the reconstruction loss function in DAE, which can preserve the intrinsic structure of the data. $\alpha$ is used to balance the importance of the clustering and reconstruction losses. After each $z_{i}$ is assigned to a cluster, a new center is set to the mean of all data points belonging to that cluster, which is defined as:

3 $$\mu_{j} = \frac{1}{{\left| {C_{j} } \right|}} \mathop \sum \limits_{{x_{i} \in C_{j} }} z_{i}$$

where $\left| {C_{j} } \right|$ denotes the cardinality of cluster $C_{j}$. It is straightforward that the embedding $z_{i}$ and centers $u_{j}$ can be iteratively optimized via mini-batch stochastic gradient descent algorithm.

### Experimental settings

For parameter settings of our DCAE model, we tune the parameter α in Eq. (2) from 0.1 to 1 with an interval of 0.1. We adopt the adaptive moment estimation (Adam) to train the network with β1 = 0.9 and β2 = 0.999. We randomly select half of the data for model training in the training process, and the remaining is used for performance evaluation. We fix the learning rate as 1e−4 and the mini-batch size for the stochastic gradient algorithm is set as 100. In order to reduce the risk of overfitting, we also add the L1-norm regularization term on the network parameters, and its corresponding regularization parameter is fixed as 5e−3. All the programs are executed on a single Nvidia K420 2 GB GPU equipped on a desktop computer, with an Intel E5-1603 CPU and 32 GB memory.

### Statistical analysis

Descriptive statistics were performed to determine patient characteristics associated with different cohorts or clusters. Characteristics of individuals presenting to different cohorts or clusters were calculated using mean and standard deviation for continuous variables (age), and frequency or proportion for categorical variables. Comparisons between the cohorts or clusters were performed using the Chi-square test for categorical variables. All statistical analyses were performed using SAS version 9.4 (SAS Institute Cary, NC).

## Data Availability

The datasets generated and/or analyzed during the current study are not publicly available due the identifiable information contained in the EMR data, but code is available from the first author on request.

## Abbreviations

CC: Chronic cough
Non-CC: Non-chronic cough
DCAE: Deep clustering algorithm with auto-encoder embedding
DAE: Deep AutoEncoders
HC: Hierarchical agglomerative clustering
CC: Consensus clustering
DC: Deep clustering
ABG: Arterial Blood Gas Test
ICD: International Classification of Diseases
NLP: Natural language processing
BN: Batch normalization
